# Study protocol: a randomized, double-blind, parallel, two-arm, placebo control trial investigating the feasibility and safety of immunoglobulin treatment in COPD patients for prevention of frequent recurrent exacerbations

**DOI:** 10.1186/s40814-018-0327-z

**Published:** 2018-08-11

**Authors:** Juthaporn Cowan, Sunita Mulpuru, Shawn Aaron, Gonzalo Alvarez, Antonio Giulivi, Vicente Corrales-Medina, Venkatesh Thiruganasambandamoorthy, Kednapa Thavorn, Ranjeeta Mallick, D. William Cameron

**Affiliations:** 1 Department of Medicine, Division of Infectious Diseases, University of Ottawa, 501 Smyth Road, Ottawa, K1H 8L6 Ontario Canada; 2 Ottawa Hospital Research Institute, Ottawa, Ontario Canada; 3 Department of Pathology and Laboratory Medicine, The Ottawa Hospital, University of Ottawa, Ottawa, Ontario Canada; 4 The Department of Emergency Medicine, The Ottawa Hospital, University of Ottawa, Ottawa, Ontario Canada; 5 School of Epidemiology, Public Health and Preventive Medicine, Faculty of Medicine, University of Ottawa, Ottawa, Ontario Canada; 6 Institute of Clinical and Evaluative Sciences, Toronto, Ontario Canada

**Keywords:** Immunoglobulin, COPD, COPD exacerbation, Pilot study, Randomized clinical trial, Feasibility and safety

## Abstract

**Background:**

Chronic obstructive pulmonary disease (COPD) is a chronic progressive inflammatory disease of the airways, associated with frailty, disability, co-morbidity, and mortality. Individuals with COPD experience increased risk and rates of acute exacerbation as their lung disease worsens. Current treatments to prevent acute exacerbation of COPD (AECOPD) are only modestly effective. New therapies are needed to improve the quality of life and clinical outcomes for individuals living with COPD and especially for those prone to frequent recurrent AECOPD. Recent research has suggested an association of gammaglobulin or immunoglobulin G levels with AECOPD and a favorable effect of an immunoglobulin treatment on the frequency of recurrent AECOPD, healthcare provider visits, treatments, and hospitalizations. However, control trials are required to confirm this apparent association and therapeutic effect. This study aims to assess if intravenous immunoglobulin (IVIG) therapy is feasible, safe, tolerable, and potentially effective in reducing the frequency of recurrent AECOPD.

**Methods/design:**

Adult COPD patients at The Ottawa Hospital (TOH) will be recruited to partake in a randomized double-blind, parallel, two-arm, placebo control trial. Eligible patients will be administered either IVIG or normal saline following 1:1 randomization and every 4 weeks for 1 year. The primary outcome of feasibility will be determined by recruitment, patient adherence, safety and tolerance, success of the follow-up procedures, and outcome measurement. The safety and tolerability will be assessed through adverse events, adherence, and study withdrawals. Efficacy trends will be investigated by assessing incidence rates of AECOPD, improvement in quality of life, and healthcare services use and cost.

**Discussion:**

The study results will inform larger studies designed to confirm a clinically significant therapeutic effect in identifiable populations which would be a major advance in the care of COPD patients.

**Trial registration number:**

ClinicalTrial.gov, NCT03018652 and NCT02690038.

**Electronic supplementary material:**

The online version of this article (10.1186/s40814-018-0327-z) contains supplementary material, which is available to authorized users.

## Background

Chronic obstructive pulmonary disease (COPD) is a chronic respiratory disease characterized by a progressive decline in lung function, shortness of breath, exercise limitation, poor health status, and increased mortality. The World Health Organization cites COPD as the third leading cause of death worldwide and estimates that it affects 65 million people [1]. COPD is a leading cause for hospital admission and readmission in North America and is thought to cost the Canadian healthcare system more than 700 million dollars annually [2].

Patients with COPD experience episodic flares of their disease. Clinically, these acute exacerbations of COPD (AECOPD) are characterized by increased cough, shortness of breath, sputum production, weakness, and worsening airflow obstruction [3]. Pathologically, AECOPD correlate with a high degree of systemic inflammation and immune system activation. As COPD severity worsens, the frequency of exacerbations increases [4]. AECOPD likely increases the progression of COPD, and additionally, it is likely that the inflammatory milieu created by AECOPD increases susceptibility to additional, recurrent AECOPD [4].

AECOPD have a significant impact on individual well-being and the healthcare system. Previous studies have shown that patients with exacerbations experience reductions in quality of life such as the ability to engage in activities of daily living, a worsening of lung function, and an increased risk for mortality both during and after the acute exacerbation period [5]. Moreover, exacerbation events often cause the patient to seek acute medical attention and admission to hospital, which drives the large healthcare costs [6]. History of hospital admission due to AECOPD is the strongest risk factor for readmission for recurrent AECOPD within 1 year [7, 8]. Hospitalization for AECOPD is also associated with lower 3-year survival as compared to COPD patients without a history of hospitalization in the previous 3 years, independent of the severity of airflow limitation [7]. As a result, reducing exacerbations has been a strong focus of research, with potential direct positive impacts to patients and the healthcare system.

Unfortunately, there is no cure for COPD, and highly effective therapies are currently lacking. The current Global Initiative for Chronic Obstructive Lung Disease (GOLD) guidelines recommend smoking cessation, exercise training, maximal bronchodilator therapy, and influenza and pneumococcal vaccinations to prevent exacerbations [9]. In patients with frequent exacerbations, chronic macrolide therapy with azithromycin [10], *N*-acetylcysteine [11], and roflumilast (a phosphodiesterase-4 inhibitor) [12] have been shown to increase the time to recurrent exacerbation. However, these therapies are only modestly effective, and patients continue to experience exacerbations while on maximal therapy. Further research into new therapeutics to prevent and reduce exacerbations is imperative. The development of newer immunomodulatory agents as adjuvant therapy to prevent AECOPD has become an area of intense investigation [13, 14].

Patients with COPD have lower immunoglobulin G (IgG) levels than patients with other lung diseases independent of oral steroid use and age [15]. Moreover, lower baseline serum IgG or gammaglobulin levels are correlated with higher rates of AECOPD [16, 17], but recurrent AECOPD still occur despite having normal baseline serum IgG as well [16]. There is no evidence of a threshold level of gammaglobulin or IgG to predict recurrent AECOPD risk, it rather appears to be a continuous, or linear relationship that extends into the “normal” range of immunoglublin G levels. From the evidence of association, it is possible that both the exacerbator phenotype and treatment cause low levels and that low levels cause a recurrent exacerbation. Neither suggests whether immunoglobulin treatment may or may not offer benefit.

Immunoglobulin for intravenous and subcutaneous infusion (IVIG and SCIG) is prepared from pooled plasma from thousands of healthy blood or plasma donors. The large donor pool ensures a diversity of antibody specificities to a wide spectrum of antigens and microbial pathogens [18]. Immunoglobulin for transfusion represents a privileged source of natural antibodies (NAb) that occur in the absence of autoimmune disease or immunization. NAb are not only an immune defense against pathogens [19, 20] but also have anti-inflammatory and immunomodulatory activities [21, 22]. Given the heightened systemic and airway inflammation in COPD, propensity for infection-triggered AECOPD, and suppressed mucosal or systemic immunity [23, 24], the anti-inflammatory, anti-infective, and immunomodulatory effects of Ig preparations could also be beneficial in this group.

We recently reported a retrospective, single-center, case series of Ig treatment as adjunctive preventative treatment for AECOPD in 14 patients [25]. Half (eight patients) had at least severe COPD by GOLD criteria. Ig treatment significantly reduced average moderate and severe AECOPD from 4.7 ± 3.1 to 0.6 ± 1.0 episodes per patient-year. Number of hospitalizations was markedly reduced from 12 in the year prior to 1 in the year following Ig treatment initiation [25]. Even though the median baseline IgG level in this study cohort was 5.9 g/L (interquartile range 4.1–7.4), and 36% had IgG less than 5 g/L, the apparent effect of Ig treatment in reducing moderate and severe AECOPD was consistent across levels of IgG. This similar effect was also demonstrated by another group in patients with demonstrated immunodeficiencies (mostly specific antibody deficiency) [26]. While this demonstrates some promise, prospective controlled studies are required to determine if Ig treatment could have any impact on the frequency of AECOPD in general. To ensure a successful definitive efficacy trial, we will perform a feasibility study to identify potential barriers and areas for improvement in conducting the study from the start to the end of the trial. We will also come up with an effect size estimate that can be used to calculate the sample size for the definitive efficacy trial.

This paper presents the rationale and describes the protocol for a pilot randomized control trial investigating the feasibility of immunoglobulin treatment in patients with a history of frequent recurrent AECOPD, or with a high risk of recurrent AECOPD. This study aims to determine if immunoglobulin treatment is safe, tolerable, and potentially effective in reducing the frequency of acute exacerbations. An effort in design will be to explore differences according to AECOPD history, underlying hypogammaglobulinemia, and comorbidity of bronchiectasis among the baseline characteristics. An effort in execution will be to draw patients from different settings, including inpatients, emergency department (ED) arrivals, and outpatients. If this study is feasible, it will inform larger studies to confirm the therapeutic effect of immunoglobulin treatment and would be a major advance in the care of COPD patients.

## Methods/design

### Study design, setting, and participants

The study design will be a pilot randomized double-blind, parallel, two-arm, placebo control trial, conducted at the clinical investigation unit (CIU) of the Ottawa Hospital Research Institute (OHRI), which is a research institute embedded in The Ottawa Hospital (TOH). TOH is an acute care university teaching hospital with over 1000 inpatient beds. We will recruit hospitalized, ED patients and outpatients with AECOPD. Due to the differences in the inclusion criteria, treating and recruiting staff, and dosing, administratively distinct protocols will be used for in- and outpatients and among inpatients for those with and without hypogammaglobulinemia. Outpatients will be identified for recruitment within the respiratory ambulatory care clinics, and hospitalized patients will be identified within the ED, and by screening patients admitted under general internal medicine or respiratory services. Patients who have provided permission to be contacted by a clinical research personnel will be approached to participate in the trial. If the participant agrees to participate in the trial by signing the consent form, screening for eligibility will commence. If no lung function testing was documented within the last 12 months, lung volume (forced expiratory volume at 1 s (FEV1) and forced vital capacity (FVC)) will be measured by a hand-held spirometer at the bedside. Blood tests and urinalysis will be sent to determine eligibility. A screening log for patients will be kept to identify the areas of improvement to enhance recruitment in future trials.

Eligible patients must be over 40 years old, have a confirmed diagnosis of COPD with a post-bronchodilator FEV1/FVC ratio of < 0.7 by spirometry within the previous 12 months, have a > 10 pack-year smoking history, and must be expected to live at least 12 months. Inpatients must have previously either visited the ED (moderate AECOPD) or been hospitalized for AECOPD (severe AECOPD). Outpatients must have a history of at least one exacerbation requiring hospitalization (severe AECOPD) or two exacerbations treated as an outpatient (moderated AECOPD) within the previous 12 months. Patients will be excluded from the study if any of the following criteria are met: known hypersensitivity to immunoglobulin product, active malignancy, transplant recipient, on immunomodulating or immunosuppressive agents apart from a short course of prednisone for AECOPD, primary antibody deficiency in need of immunoglobulin therapy, IgA deficiency (IgA < 0.1 g/L), nephrotic syndrome, currently on IVIG or SCIG, or current pregnancy.

### Interventions

Random 1:1 allocation will be stratified by outpatient or inpatient groups and further by baseline IgG < 7 g/L or IgG ≥ 7 g/L. In the outpatient group, the treatment arm will receive doses of 0.5 g/kg, and the control arm will receive normal saline at 5 mL/kg. Doses of IVIG and normal saline will be administered to the respective patient populations on the day of randomization and then every 4 ± 1 weeks for 44 weeks (total of 48 weeks). In the inpatient group with low baseline IgG, the treatment arm will receive doses of 0.8 g/kg, and the control arm will receive normal saline at 8 mL/kg. Treatment arm of inpatients with normal IgG levels will receive doses of 0.5 g/kg, and the control arm will receive normal saline at 5 mL/kg. A maximum dose of 80 g/dose of IVIG 10% (Gamunex or CBS IGIV-nex or Privigen or Octagam depending on availability at the blood bank) will be given to patients randomized to treatment arms. Consistent IVIG brand or formulation is routinely maintained for individual patients according to the first dose received and for subsequent doses. For control patients, normal saline will consist of 0.9% NaCl, and a maximum dose of 800 mL will be given.

For all patients, respective doses of IVIG and normal saline will be administered over an infusion time of 4–6 h by a nurse in the CIU. Doses will be determined based on patient’s weight at the time of enrolment and will be consistent throughout the study unless there is a change in weight of at least 10%, in which case, the dose will be adjusted to the new body weight. All patients will continue to receive standard treatment for COPD as directed by their regular treating physician.

Participants may withdraw from the study at any time and for any reason; however, they will be encouraged to stay on their assigned study medications in the absence of illness- or treatment-related adverse events. All patients will be followed up for adverse events and outcomes for 1 year from randomization. The sponsor (OHRI), the qualified investigator, or a treating physician may withdraw the participant at their discretion or in the event of intercurrent illness, intolerance to the study treatment, adverse events, pregnancy, protocol violation, or administrative reasons. All participants discontinued due to an adverse event will be followed until the event resolves or becomes chronic or stable. Appropriate medical care will be provided until signs and symptoms have remitted or stabilized or until abnormal laboratory findings have returned to acceptable or pre-study limits.

After the study completion, patients will continue their clinical care with their primary care provider or specialist, as prior to and during the study period. Patients may elect to stay on IVIG treatment by prescription according to the judgment of the treating physician.

### Allocation of study treatment and blinding

Each participant will receive a unique participant identification (ID) number. The trial statistician will designate another statistician to prepare all randomization schemes. A computer-generated randomized allocation output will be given to CIU nurse in an unblinded fashion. The CIU nurse will send a request to TOH blood bank to issue IVIG product with the participant ID number on it at first and all subsequent visits. Only the blood bank staff and the CIU nurse will have knowledge of the treatment allocation. The CIU nurse will prepare IVIG or normal saline infusion kit, and these will be administered in a concealed manner. In order to minimize the selection and ascertainment biases, physicians, investigators, and research staff will be blinded to the randomization schemes and treatments administered. Unblinding should only occur under exceptional or emergency circumstances where death or life-threatening complications require knowledge of the study treatment allocation. This information will allow the investigator to make an informed decision regarding the treatment and management of the participant.

### Outcomes

The primary outcome of interest is the feasibility, which is determined by the following:

1. Recruitment—ability to recruit on an average of four patients per month.
2. Adherence—at least 80% of patients adhere to 80% of allocated treatment and protocol
3. Retention of subject—at least 80% of patients remain followed up to 12 months.

All secondary outcomes will be ascertained during the week 4, 12, 24, 36, and 48 visits, as well as by monthly in-person or phone assessments by our study coordinator. Study participants will be encouraged to call the study coordinator anytime if they experience any adverse events. Secondary outcomes are as follows:

1. Safety: This will be assessed by documentation of adverse events in patients treated with Ig treatment versus control. Known adverse events associated with the treatment include headache, chills, fever, weakness, nausea arthralgia, malaise, urticaria, leukopenia, tachycardia, hypotension, anaphylaxis, localized injection reactions, and thrombotic events (arterial or venous). All adverse events will be graded by the investigator using a 5-point scale (Table 1) and scored as to attributability to study treatment.
2. Tolerability: The proportion of patients able to complete the treatment in the experimental arm during the study period versus in the control arm.
3. Efficacy trends (this study is not powered to show significance):
  - AECOPD outcomes will be measured by (i) percentage of patients with one or more AECOPD episode, (ii) time from randomization to the first moderate or severe AECOPD, and (iii) annualized moderate or severe AECOPD rates (number of exacerbations/12 months). AECOPD will be ascertained by monthly follow-up phone calls to detect outpatient treatment for exacerbations. AECOPD is defined as a complex of increased or new onset respiratory symptoms of more than one of cough, sputum, wheezing, dyspnea, or chest tightness, with a duration of at least 3 days requiring treatment with prescribed antibiotics and/or systemic steroids [3]. Research staff will determine if the patients have met this definition of exacerbation, or if the patient was treated with antibiotics or steroid medication for an AECOPD.
  - Health status will be measured by the validated St. George Respiratory Questionnaire (SGRQ) and COPD Assessment Test (CAT) scores.
  - Quality of life will be measured by the validated quality of life measurement tool, EuroQol EQ-5D-5L.
  - FEV1 and FVC measurements will be carried out with a hand-held spirometer and recorded in liters and as a percent of their predicted value, using the NHANES III reference standards for predicted values.
  - Health services use is defined by non-study physician visits, ED, and hospital admissions over the 12-month study period. Research staff will ascertain this by reviewing the patient’s medical record and confirming any hospital visits and cause with the patient and his/her primary care provider.
  - Healthcare system cost: We will measure the cost of health services use and the intervention. Intervention cost includes medication, staff, and equipment cost. The data will be captured by clinical report forms, TOH warehouse database, and a cost questionnaire (Additional file 1) designed for this study, which will be collected at weeks 12, 24, 36, and 48.

**Table 1:** Five-point grading scale of adverse events

1 = mild	Discomfort noticed but no disruption of normal daily activity
2 = moderate	Discomfort sufficient to reduce or affect daily activity
3 = severe	Inability to work or perform normal daily activity
4 = life threatening/disabling	Represents an immediate threat to life
5 = death	Related to adverse event

In order to avoid bias and inaccurate reporting, a blinded outcome adjudication committee will be formed to assess the outcomes documented by trial investigators as well as outcomes not captured by the investigators but by other healthcare professionals. There will be two independent blinded experts appointed for the outcome adjudication process. The outcome adjudication committee will assess the outcome data every 12 months until study closure.

### Patient recruitment

The study description will be distributed to the members of the Division of Respirology who are working in ambulatory care clinics and members of general internal medicine and ED who are likely to admit patients with AECOPD. Study posters will be placed in the clinic workspaces, ED consultant areas, and on the inpatient wards. Study coordinators will identify potentially eligible patients through a daily report of admission diagnosis and/or from attending physicians. The candidate who has provided permission to be contacted by clinical research personnel will be approached to participate in the trial.

### Study visits

Participant study visits are detailed in Table 2. Study visits will include a pre-treatment evaluation to obtain informed consent and demographic information as well as assess eligibility for participation. At a baseline study visit (week 0), treatment allocation and the first infusion of IVIG or normal saline will take place. Infusion of IVIG or normal saline will continue on monthly treatment visits (± 6 days). Assessment of primary and secondary outcomes will take place as indicated in Table 2. Adverse events, a review of concomitant medications, and the CAT questionnaire will be assessed on follow-up via monthly telephone calls. Note that the total study period is planned for 2 years (1-year accrual period and 1-year follow-up of each subject). All patients will be followed up until the end of the study closure or until the last recruited subject is followed for 12 months from the time of enrolment in the study.

**Table 2:** Study visits and participant timeline

	Enrolment	Week 0	Week 4	Week 8	Week 12	Week 16	Week 20	Week 24	Week 28	Week 32	Week 36	Week 40	Week 44	Week 48	Follow-up
Written informed consent	X														
Demography	X	X													
Eligibility verification	X	X													
Allocation		X													
Complete physical exam	X														
Vital signs including Sp02	X	X	X	X	X	X	X	X	X	X	X	X	X	X	
Height	X														
Weight	X	X	X	X	X	X	X	X	X	X	X	X	X	X	
Urine pregnancy test^1^	X														
Pulmonary function test or spirometry	X	X^a^	X		X			X			X			X	
Target physical exam			X		X			X			X			X	
Immunoglobulin profiles and other hematological and biochemical tests^2^	X		X^b^		X^b^			X^b^			X^b^			X^b^	
CT chest, chest X-ray, sinus X-ray^3^	X	X													
ECG, nasopharyngeal swab, sputum culture^4^	X	X													
Medical history	X	X	X	X	X	X	X	X	X	X	X	X	X	X	X
Adverse event assessment	X	X	X	X	X	X	X	X	X	X	X	X	X	X	X
Concomitant medications	X	X	X	X	X	X	X	X	X	X	X	X	X	X	X
Infusion of Ig or normal saline		X	X	X	X	X	X	X	X	X	X	X	X	X	
EQ-5D-5L questionnaire		X	X		X			X			X			X	
SGRQ questionnaire^5^		X	X		X			X			X			X	
COST Questionnaire			X		X			X			X			X	
CAT Questionnaire		X	X	X	X	X	X	X	X	X	X	X	X	X	X
Hunch survey				X										X	X

All laboratory tests will be performed at the hospital

^1^Only women of child-bearing potential will have a pregnancy test conducted at the screening visit

^2^α1-antitrypsin will be measured once at time of enrolment or allocation or at week 4 visit if this has not been performed or documented in the participant’s medical record in the past

^3^CT of chest (within the last 1 year) and chest X-ray (during this admission) data will be collected and recorded. If patient has not had CT of chest within the past year or chest X-ray during this admission, it will be requested and ordered by PI or Co-PI. If sinus X-ray results are available within the last year they will be recorded. If participant has not had sinus X-ray completed within the last year it will not be requested or ordered unless participant is symptomatic or suspected to have acute or chronic sinusitis

^4^ECG results will be recorded on CRF if available within the last year. If ECG has not been performed within the last year, an ECG will be ordered and conducted by the CIU nurses. Nasopharyngeal swabs and sputum culture data will be recorded if available within the last year. Microbiological studies of respiratory samples (nasopharyngeal swab for viral testing and sputum culture) will not be performed if not already available at time of screening unless clinically indicated

^5^SGRQ 4 weeks will be used at allocation and week 4 visit while SGRQ 3 months will be used at week 12, week 24, week 36, and week 48

^a^Results of PFT in the past 12 months. If not available, conduct a bedside spirometry testing

^b^Excluding IgG subclass testing

### Sample size

For feasibility, safety, and tolerability, we will aim to recruit 16 outpatients and 48 inpatients. As this is a pilot study, we will not power the study to show efficacy in exacerbation reduction. Informed by our current clinic patient volume, we estimate that we can feasibly recruit 64 patients in the 2-year study period and have chosen this as the target sample size for this pilot trial. However, if we conservatively assumed that 30% of severe COPD patients develop exacerbation episode within 12 months [10], 59 patients would be required on each arm to have 80% power to detect 20% reduction in AECOPD within 12 months. Based on our case series [25], there was an 86.2% apparent reduction in the average number of exacerbations in 1 year in patients treated with immunoglobulin. This data may be used to estimate the sample size for a future trial of similar design.

### Data collection, management, and analysis

All data management and statistical analyses will be performed by the Ottawa Methods Centre at the OHRI. Study personnel will collect and enter the data on electronic case report forms, SQGR, EQ-5D-5L questionnaires, and patient cost questionnaire designed to suit this study. Case report forms will be entered into a secure database, which will be password protected and stored behind the research institution’s secure firewall. All personal health information will be kept confidential, unless release is required by law.

We will use descriptive statistics including number and proportion, means standard deviations (± SD), and medians (interquartile range) to describe the baseline characteristics of our study cohort. Descriptive analysis of recruitment, adherence, and retention of participants will also be performed in order to use this information to make changes in the definitive clinical trial.

Adverse events and tolerability will be summarized using point estimates with 95% CI for the intervention and control groups. All statistical analysis will be performed using SAS 9.3 by SAS Institute Inc., Cary, NC, USA.

Since this study is not powered to evaluate the efficacy, the changes in FEV1 value, the SGRQ and EQ-5D-5L scores, and the health service utilization will be described but not comparatively analyzed by the group as a primary outcome. AECOPD rates will be calculated on a per person/per year basis and described. To inform sample size calculations, we will use the following data obtained from this study: (1) percentage of patients with at least one recurrent AECOPD in 1 year, (2) time to first recurrent AECOPD and first recurrent hospitalization due to AECOPD, and (3) 1-year incidence of AECOPD overall and in intervention and control groups.

Routine quality control will be completed by the qualified investigator or designee at the CIU to ensure that the study is being run according to the protocol. A Data Safety Monitoring Board (DSMB) will also be appointed to assure that all participants are not exposed to unnecessary or unreasonable risks and that the study is being conducted to the highest scientific and ethical standards. DSMB meeting will occur every 6 months until the study closure. The study will be terminated if necessary for the safety of participants upon DSMB recommendation.

### Safety

An adverse event is any untoward medical occurrence in a participant administered an investigational product, which does not necessarily have to have a causal relationship with the treatment. The investigator or designate will collect a detailed description of the adverse event if the participant has experienced a new event or change in existing medical condition or symptom and is either observed or volunteered, serious, or non-serious. All serious and/or significant unexpected, related, or possibly related adverse events, regardless of treatment group, will be reported to the institutional research ethics board and Health Canada within 72 h in writing.

### Dissemination

We will communicate with relevant parties such as investigators, institutional research ethics board, trial participants, trial registries, and Health Canada in writing for any changes in the protocol including eligibility criteria and outcomes.

The results of this study will be published in an open access peer-reviewed journal, and key findings will be presented at scientific meetings and investigator meetings for discussion and design of future trials. Participants will not be identifiable in any publications or presentations resulting from this study. The results of this study will be released to patients, participating physicians, referring physicians, supporting (funding) biopharma companies, and general medical community regardless of magnitude or direction of effect.

### Trial status

Participants are being recruited.

## Discussion

- If feasible, this pilot randomized controlled trial may help guide a larger, definitive, pragmatic randomized controlled trial to determine the effectiveness of Ig treatment in patients with frequent exacerbations.
- Although outcome trends may be demonstrated, this study is not adequately powered to show significance with regard to IVIG efficacy in treating AECOPD.
- This and future trials may permit additional measurements of explanatory interest as to possible mechanisms of preventative efficacy.

## Additional file

Additional file 1: Cost Questionnaire. (PDF 335 kb)

## Abbreviations

AECOPD: Acute exacerbation of COPD
CAT: COPD assessment test
CIU: Clinical investigation unit
COPD: Chronic obstructive pulmonary disease
DSMB: Data safety monitoring board
ED: Emergency department
EQ-5D-5 L: EuroQol-5 dimension-5 levels
FEV1: Forced expiratory volume at 1 s
FVC: Forced vital capacity
GOLD: Global initiative of chronic obstructive lung disease
ID: Identification
IgA: Immunoglobulin A
IgG: Immunoglobulin G
IVIG: Intravenous immunoglobulin
Nab: Natural antibody
NHANES: National Health and Nutrition Examination Survey
OHRI: The Ottawa Hospital Research Institute
SCIG: Subcutaneous immunoglobulin
SGRQ: St. George Respiratory Questionnaire
TOH: The Ottawa Hospital
