# Supplementary material for: Study protocol: a randomized, double-blind, parallel, two-arm, placebo control trial investigating the feasibility and safety of immunoglobulin treatment in COPD patients for prevention of frequent recurrent exacerbations
Source: Pilot Feasibility Stud. 2018 Aug 11;4:135. doi: 10.1186/s40814-018-0327-z (PMC6087014; doi:10.1186/s40814-018-0327-z)
Supplement: Supplementary file 1 — Cost Questionnaire. (PDF 335 kb) [file 40814_2018_327_MOESM1_ESM.pdf]

Cost Questionnaire

Study title: Ig PRx in AECOPD
Screening Number: _______________
Participant Study ID: ____________________ Participant Pseudo Initials ___-___-___
Study Start Date(yyyy/mmm/dd): ____________________
Call Date (yyyy/mmm/dd): ___________________ Number of week of study: ___________

Resource use

1. Could you please tell me how many times you have used any of the following services in the last 3 month?

	Have you used this service? Please write Yes or No	If yes, how many times have you used this service in the last 3 months?
GP or family physician visits		
Walk-in clinic		
Specialist office visits		
Home care nurse visits		
Social worker visits		
Other health visitor visits e.g. physiotherapist, occupational therapist, psychologist, etc.		
Home help such as personal support worker		
Any other professional visitor or service (please specify)		

2. Have you been admitted to hospital in the last 3 month?
   ___ Yes   How many times? _____
   ___ No (go to question 4)

3. Think about your hospitalization(s)
   Stay #1
   a. When was date admitted? ______________
   b. When was date discharged? ____________
   c. Name of hospital and town ______________
   d. Were you admitted to ICU? _______________

e. If you were admitted to ICU, how many days were you in ICU? ______

Stay #2

a. When was date admitted? _____________
b. When was date discharged? _____________
c. Name of hospital and town _______________
d. Were you admitted to ICU? _______________
e. If you were admitted to ICU, how many days were you in ICU? _______

Stay #3

a. When was date admitted? ______________
b. When was date discharged? _____________
c. Name of hospital and town ______________
d. Were you admitted to ICU? _______________
e. If you were admitted to ICU, how many days were you in ICU? ______

Stay #4

a. When was date admitted? _____________
b. When was date discharged? _____________
c. Name of hospital and town _______________
d. Were you admitted to ICU? _______________
e. If you were admitted to ICU, how many days were you in ICU? _______

Medication

1. Have there been any new prescription or non-prescription medications in the past month?
   __ Yes
   __ No

   If yes, please fill in the table below

**Medication name and dosage**	**How much money did you pay from your own pocket?**	**How often do you take this medication?**	**Are you currently taking this medication?**

2. Have there been any medications you stopped taking in the past month?
   __ Yes
   __ No
   If yes, please list name and stopped date
   ____
   ____

Employment

1. Do you do any work as an employee or on a government sponsored training scheme, as self-employed/freelance, or in your own/family business?
   ___ Yes, full time
   ___ Yes, part time
   ___ No

2. If no, are you
   ___ Retired?
   ___ Student?
   ___ Looking after home/family?
   ___ Permanently sick/disabled?
   ___ None of the above?

3. If you are in paid employment, how many days have you had off work in the last month on account of your health?
   __________________ days

Caregiver

1. Do you receive help with your daily activities from a relative or friend?
   ___ Yes
   ___ No (end of questionnaire)

2. If yes, on average, how much time per day/per week do they spend?
   ______________________ hours/day
   ______________________ days/week

3. What would that person have been doing as their main activity if they had not been helping and/or caring for you?
   ___ housework
   ___ childcare
   ___ caring for a relative or friend
   ___ voluntary work
   ___ leisure activities
   ___ attending school or university
   ___ on sick leave
   ___ paid work
   ___ other (please specify) ______________________

4. What is your caregiver's occupation?
   ____
